# Observation and Institutional Ethnography: Helping Us to See Better

**DOI:** 10.1177/10497323211015966

**Published:** 2021-06-07

**Authors:** Sarah Balcom, Shelley Doucet, Anik Dubé

**Affiliations:** 1 University of New Brunswick, Fredericton, New Brunswick, Canada; 2 University of New Brunswick, Saint John, New Brunswick, Canada; 3 Université de Moncton, Moncton, New Brunswick, Canada

**Keywords:** qualitative, nursing, research design, methodology, ethnography, institutional ethnography

## Abstract

Observation is a staple data collection method, which is used in many qualitative approaches, including both traditional and institutional ethnographies. While observation is one of the most used data collection methods in traditional ethnography, less is written about its use by institutional ethnographers. Institutional ethnography is an approach to social research where the aim is to explicate how peoples’ every activities are coordinated or ruled by different institutions. In this article we explore uses of observation as a data collection method, focusing on its use in institutional ethnography. We use examples from the health care literature to show how observation can be beneficial and help institutional ethnographers see better.

You can see a lot by just observing.

—Yogi Berra

## Introduction

Observation, a staple data collection method, has been used for over century and is useful to researchers in a variety of ways (Conroy, 2017). For example, it enables researchers to collect data about expressions of feelings, interactions between people, and time is spent on various activities (Schmuck, 1997). While observation is arguably one of the most used data collection methods in traditional ethnography, less is written about its use by institutional ethnographers. In this article, we discuss observation and its usefulness in health care research. We then describe both institutional and traditional ethnography, focusing on the differences between these two approaches. Although there are many similarities between institutional and traditional ethnography, there are also many differences, particularly related to their theoretical underpinnings and goals of data analyses. These differences have implications for how institutional and traditional ethnographers use and prioritize observation, along with other data collection methods. Institutional ethnographers generally do not prioritize observation as a data collection method as highly as traditional ethnographers do. Researchers use observations to understand what people are doing locally. Institutional ethnographers, however, aim to explicate how peoples’ local activities are coordinated by different social institutions (Rankin, 2017). Consequently, institutional ethnographers place a higher value on interviews and textual analysis, which bring the rule of these institutions into view (Rankin, 2017). As Smith (2006) writes, “texts are of central importance to [institutional ethnography] because they create this essential connection between the local of our (and others’) bodily being and the translocal organization of the ruling relations” (pp. 118–119). To conclude, we address some concerns that appear in the literature regarding the use of observation as a data collection method in general, and how these translate for institutional ethnographers. We use examples from health care literature to argue how observation is still beneficial and can help institutional ethnographers see better.

## Observation as a Data Collection Method

According to Marshall and Rossman (1989), observation is “the systematic description of events, behaviors, and artifacts in the social setting chosen for study” (p. 79). Several researchers argue that today observation is an underused and is less favored than interviews as a data collection method in qualitative health care research (Conroy, 2017; Dympna, 2006; Mulhall, 2003). This is surprising because health care providers themselves are trained to be “good observers” and often view observation as essential for their practice. Nightingale (1860/1969) recognized the importance of observation as a skill for nurses. In “*Notes on Nursing*,” she wrote:

The most important practical lesson that can be given to nurses is to teach them what to observe—how to observe—what symptoms indicate improvement—what the reverse—which are of importance—which are of none—which are evidence of neglect—and of what kind of neglect. All this is what ought to make part, and an essential part, of the training of every nurse. (p. 105)

Health care providers often observe their patients’ verbal and nonverbal behaviors when they complete their physical assessments and use these observations to enhance their overall data collection. For example, observations can cue health care providers to ask their patients particular questions. A health care provider might ask about a long sternotomy scar and learn about a patient’s bypass surgery and struggles to quit smoking. Observations can also be used to help interpret things that people verbally report or to further understand peoples’ experiences. As Mulhall (2003) argues, it assists researchers in similar ways, such as by allowing researchers to see what actually happens in a particular, local setting.

Observation is frequently used in qualitative approaches and is either structured or unstructured (Mulhall, 2003). Structured observation requires investigators to unobtrusively record their participants’ physical and verbal actions from afar (Salmon, 2015). It is most suitable for positivistic research and is a useful data collection method when the research question and information needed are defined (Mulhall, 2003). There are examples of structured observation in health care research. For example, Duxbury et al. (2010) used structured observation to code nurse–patient behaviors and interactions during medication administration; and Montgomery et al. (2020) used structured observation to code motor performance in children born preterm.

Researchers who use unstructured observation consider the spontaneous behaviors and interactions of people engaging in their daily activities (Mulhall, 2003). This data collection method is frequently used in traditional ethnographies and is a good fit with the naturalistic paradigm (Mulhall, 2003). O’Connell Davidson and Layder (1994) explain how traditional ethnography “belongs to the tradition of’ ‘naturalism’, which centralizes the importance of understanding the meaning and cultural practices of people from within the everyday settings in which they take place” (p. 165). According to Bisaillon and Rankin (2012), institutional ethnography is “a critical research strategy located within a post-positive paradigm” (p. 1). Like positivism, post-positivism is rooted in the belief that there is a knowable world (Guba & Lincoln, 1994). Post-positivism, however, holds that researchers’ must reflect on their biases because these can taint the research process (Guba & Lincoln, 1994). Institutional ethnographers can use unstructured observations to begin knowing people’s everyday activities. For example, Corman (2017) completed more than 200 hours of observation to see how paramedics work in and on their ambulances. There is, however, a limit to the knowable world in a particular setting, so institutional ethnographers need to use other data collection methods, like interviews and textual analysis, to trace threads of coordination to other people’s activities located elsewhere. As Smith (2006) explains, while institutional ethnographers begin “where people are and proceeds from there” in an outward direction (p. 3).

## Traditional Ethnography

Although both institutional ethnographers and traditional ethnographers use observation, there are differences between these two qualitative approaches that determine how observation is used and prioritized. Most researchers are familiar with traditional ethnography when compared to institutional ethnography (Conroy, 2017). Traditional ethnography became a popular approach at the end of the 19th century, during the era of western colonialism and is one of the oldest qualitative methodologies (Rashid et al., 2015). Traditional ethnographers seek to develop a “formal description of foreign people, their habits, and customs” (Almagor & Skinner, 2013, p. 2). When people think about traditional ethnography, they often picture researchers who live among and observe a particular cultural group to learn about specific aspects of their daily lives. Frank Hamilton Cushing is a well-known early traditional ethnographer who spent four and a half years as a participant observer with the Zuni Pueblo people around the year 1879 for a study for the Smithsonian Institute’s Bureau of Ethnology (DeWalt & DeWalt, 2002). Traditional ethnographers often rely on observation because it allows them to see what a particular group in a particular setting is doing at a particular time (Conroy, 2017). Although many researchers agree that traditional ethnography belongs to the tradition of “naturalism” (O’Connell-Davidson & Layder, 1994), some researchers are critical of traditional ethnographic descriptions (Hammersley, 1990). Hammersley (1990) argues these descriptions become realistic because the phenomena of interest are described in a single, objective way (Hammersley, 1990).

Historically, traditional ethnographies often focused on distinct cultural groups, such as the Zunis Pueblo people. According to Morse (2016), early ethnographic methods were learned “in the apprentice system, by doing it and by staying in the field until you got it right” (p. 875). Traditional ethnography remains a somewhat flexible methodology and appeals to many modern researchers seeking to understand societal interactions and experiences, among other topics. Today, traditional ethnographies may focus on an aspect of a group’s life, such as health care, or different levels of experiences among populations living in society.

## Institutional Ethnography

Institutional ethnography is a research approach that was developed during the 1970s and ‘80s by Canadian sociologist, Dorothy Smith, in her work to more accurately study womens’ experiences. She found much of the existing authoritative knowledge of this period subordinated the knowledge women had about their own experiences. This authoritative knowledge was often the knowledge of male scholars and other experts, thus did not give authority of knowing to men and women equally. This was particularly problematic because women often used this “authorized” knowledge to explain their experiences even when these explanations were not accurate.

Smith envisioned “an alternate sociology, a sociology that was not confined to a particular category of people” (Smith, 2005, p. 1). She wanted a way to study people’s actual experiences, as they are for them; and validate the knowledge and understanding people have about their own lives. This is significant to health care contexts, where traditionally the male-dominated profession of medicine authorized health care knowledge, dominating over other health professions, such the female-dominated profession of nursing. Until the early 20th century, for example, nursing professionals were taught by physicians, which was problematic because their authorized medical model did not always match nursing professionals’ experiences with their patients.

Smith drew from feminism and Marxism; and sought to develop a sociology that equally represented all people; a sociology in which anyone, regardless of their gender, could participate as a seeker of knowledge (Campbell & Gregor, 2002). Campbell and Gregor (2002) assert that “[t]he claim made for institutional ethnography is that it offers a knowledge resource for people who want to work towards a more equitable society” (p. 103). Institutional ethnography allows investigators to use peoples’ everyday experiences as their entry-points into uncovering how institutions organize and rule their lives (Devault, 2006). It is an approach to social research where the aim is to collect data which explicates how people’s daily activities in a particular, local setting are ruled by larger social institutions located elsewhere (Ng et al., 2013). For example, health care providers’ daily activities with patients are organized by decisions made by administrators in offices far away from the patients’ bedsides. Recently, researchers have successfully used institutional ethnography to generate understanding of many health care issues, such as patient satisfaction, patient-centered care, nurses’ stress, and workplace mental health (Malachowski et al., 2016; McGibbon et al., 2010; Rankin, 2003; Rankin & Campbell, 2006; Rankin & Campbell, 2009; Townsend el al., 2003).

## Similarities and Differences Between Institutional and Traditional Ethnographies

There are many similarities between institutional and traditional ethnographic studies. For example, institutional and traditional ethnographers use similar data collection methods, including observation (Campbell & Gregor, 2002). However, the goals of traditional and institutional ethnographers are different; and this has implications for how data collection methods are used and how data are prioritized/analyzed between these two approaches.

As mentioned above, traditional ethnographers seek to describe, from an insider’s understanding or perspective, the experiences of a social or cultural group or an aspect of social life located within a particular setting (Fetterman, 1989). They aim to reveal tacit knowledge of this particular group about their culture and/or social experiences (Loiselle et al., 2013). This is the knowledge that is so widely accepted by a group that its members do not talk about it and may not even be aware of (Loiselle et al., 2013). Traditional ethnographers also worry about misinterpreting their data; and usually triangulate data collected from different methods, for example, document retrieval, observation, and interviews, to increase the accuracy of their research. In triangulation, data collected by different methods are compared to increase the validity of the research (Fetterman, 1998).

Institutional ethnographers also want to collect data that display insiders’ knowledge (Campbell & Gregor, 2004; Tummons, 2017). They have, however, a different intent for their data. The ultimate purpose of an institutional ethnography is not to produce an account of or from those insiders’ perspectives, but to explicate the often invisible social relations that rule people’s everyday. Institutional ethnographers are interested in “how things work” and “how they are actually put together” as opposed to “what happens” (Kearney et al., 2019). According to Quinlan (2009), “An institutional ethnographer’s starting point is the actualities of people’s everyday experience; their end point connects the actualities to the social organization that governs the local setting” (p. 628). Consequently, institutional ethnographers use their data to trace back and describe these social relations that exist beyond people’s everyday experiences in their local setting but connect them to distant ruling institutions. This has important implications for how institutional ethnographers prioritize different data collection methods. While traditional ethnographers may rely primarily on their observations of people’s behavior in a local setting, institutional ethnographers cannot. Observations in local setting alone will not reveal to them how institutions exert their rule from afar.

Institutional ethnographers thus prioritize interviews and textual analysis over observation to gain information about distant ruling institutions. They consider “texts” to be essential to both the existence and ruling of institutions (Smith, 2001). Texts are the material forms of words, including images and sounds that are replicable (Smith, 2006; Turner, 2006). Books, radio announcements, photographs, and bus tokens are all examples of texts (Smith, 2006). When a person “activates” a text in a particular setting, he or she becomes connected to other people and processes taking place and organized elsewhere (Smith, 2006; Turner, 2006). According to Smith (2006) “institutional discourse is set in texts . . . texts are of central importance to IE because they create this essential connection between the local of our (and others’) bodily being and the translocal organization of the ruling relations” (pp. 118–119).

## Institutional Ethnography and Observation

Smith (2001) writes, “exploring how texts mediate, regulate and authorize peoples’ activities in modern societies expands the scope of ethnographic method beyond the limits of observation” (p. 159). Although, through this quote, Smith makes it clear that interviews and the analysis of texts are preferred method of data collection; observation does provide some data that may be different and informative. Observation makes it possible to confirm whether what people say they do and what they actually do match up. It is important to note that both accounts (what people perceive that they do and what they do) provide information, but the information is different. The following two examples demonstrate how observation can add to the data provided by interviews and textual analysis. These examples consider the experiences of two different health care professionals: a physician and a registered nurse. The first example shows how observation can reveal what a physician actually does when they “collaborate” with a team. The second example explains how observation reveals how a registered nurse actually activates a text during their daily work activities.

In an interview, a physician may say “I collaborated with a team,” but the actualities of what they did are missing. What activities belong to the concept “collaboration?” What does “collaboration” mean to this physician? What does it mean to their colleagues or in the hospital where they work? What does it mean to their professional association? What does it mean to the other members of the “team”? As is evident, when interviewing participants, people use language that can make their actual activities unclear. Campbell and Gregor (2002) caution that professional and conceptual language often conceals what people really do. The term “collaboration” is conceptual and may be made even more vague by being part of an institution’s professional or rhetorical language. Consequently, it blankets or covers up what the health care provider’s actual activities are. Observation can help reveal the steps the health care provider took or the texts that were activated to make this collaboration happen. As Diamond (2006) argues, observation enhances institutional ethnography’s goal of connecting people’s activities in a local setting, such as a hospital unit, to the activities of people elsewhere and larger institutions.

Similarly, in interviews, people may not fully explain how they, and those around them, activate texts in their workplaces. To illustrate this, in an interview a registered nurse may say, “I administer medications.” The registered nurse may explain, “I use my workplace’s medication administration policy each time I administer my patients’ medications.” This likely does not mean that this register nurse accesses and reads their workplace’s medication administration policy each time they need to administer medications. It does mean this registered nurse knows about their workplaces’ medication administration policy, but may not mean they follow it. Observations allow researchers to see what texts are used in a particular setting (e.g., do they access the medication administration policy?) and what actually happens (e.g., how do registered nurses administer medications?), which provides context for data gathered through interviews or textual analysis. Maybe this registered nurse does not know how to find the medication administration policy through their workplace’s intranet. Textual analysis allows institutional ethnographers to understand policies and other documents, but observation lets them “see” if and how people “activate” these texts locally in their workplaces.

One of the distinctive features of institutional ethnography is termed standpoint, which places a focus on the knowledge of people as opposed to the overarching explanations of researchers (Tummons et al., 2015). Kragelund (2013) recommends researchers use “obser-views” or observations immediately followed by interviews. Through “obser-views,” questioning may become a catalyst for informants’ reflection on the actions they completed (Kragelund, 2013). Kragelund’s (2013) “obser-views” may provide a way for institutional ethnographers to both locate their informants’ standpoint and situate their research “looking up from where [they] are” (Smith, 2006, p. 5) within the institution.

### Challenges and Opportunities for Observation With Institutional Ethnography

There are many challenges, as well as opportunities, when using observation with IE (Dympna, 2006; Mulhall, 2003). The main challenges include the presence of the researcher, time commitment, field site access, selective reporting/researcher biases, and informed consent/deception (Dympna, 2006; Mulhall, 2003). Each of these issues have been discussed and debated in nursing and other literature. They will only be discussed in this article as they relate to the use of observation in institutional ethnography, which may be different from other research approaches. Opportunities related to each challenge are also discussed.

#### The researcher’s presence

Researchers often worry about how their presence will affect peoples’ activities at their field sites. Campbell and Gregor (2002) argue that traditionally “the researcher’s presence” has been treated “as a problem that must be overcome” (p. 14). Traditional ethnographers worry about how their presence will change people’s activities and often triangulate their observational data with other data, such as interviews, to verify their “trueness” and give them evidential weight. Institutional ethnographers, however, are interested in how their observations in local settings occur and are organized and connect back to ruling institutions. Thus, the changes an institutional ethnographer’s presence creates in people’s activities becomes part of the analysis. Institutional ethnographers often represent ruling institutions, such as universities, and through their research, their presence becomes another social relation that exists in people’s local settings.

#### Time commitment

Concerns have been raised that observational research can be too time-consuming. Above, for example, we mention how Frank Hamilton Cushing spent four and a half years observing the Zuni Pueblo people (DeWalt & DeWalt, 2002). This amount of time and quantity of observational data is not needed for most institutional ethnography studies; in fact, observational data may not prolong time spent at a field site. In a discussion article, Bisaillon and Rankin (2012) discribed their experiences using institutional ethnography as a research method, working independently of each other and on separate projects. They both reflected on how their presence, just “waiting,” at field sites for interviews with people provided them with impromptu opportunities to collect observational data; which helped support and better inform their interviews (Bisaillon & Rankin, 2012). Bisaillon and Rankin (2012) explained how the process of waiting often had them “sitting in the same chairs” as the people they interviewed. Their observational data did not “add time” to their research projects because they used time that needed to be spent at their field sites for interviews anyway.

#### Field site access

Oftentimes researchers have difficulty gaining access to field sites, particularly hierarchal government-run institutions (Taber, 2010). For example, Taber’s (2010) research focused on the everyday experiences of women working in the military. She met with resistance when she tried to observe military women’s groups (Taber, 2010). She reflected afterwards that although her experience was frustrating, it encouraged her to deepen her understanding of institutional ethnography so she could adapt her original approach (Taber, 2010). Her experiences with her application to observe the women’s group also made her reflect on how inflexible the military’s processes are. To avoid frustrating experiences, Bisaillon and Rankin (2012) encourage researchers to reflect on their field sites and try to anticipate challenges, such as access, before they arise. They also recommend that researchers remain flexible and open to unexpected opportunities to collect data, such as those presented while “waiting” (Bisaillon & Rankin, 2012).

Bisaillon and Rankin (2012) both needed to make amendments for ethical approval from their respective universities to gain access to new field sites during their research. The work of ethics committees is itself text-based; and receiving ethical approval may be challenging for institutional ethnographers (Campbell & Gregor, 2002). Often institutional ethnographers do not know their interview schedules and other information required for an ethics review (Campbell & Gregor, 2002). This is a challenge for many qualitative researchers. Institutional ethnographers need to clearly explain the particulars of their research approach, so ethics review boards understand why they may need to make changes to their prospective plans as their research progresses (Campbell & Gregor, 2002).

#### Bias and selective reporting

With other qualitative approaches, such as traditional ethnography, observation is sometimes seen as an alternative to self-reports (Loiselle & ProfettoMcGrath, 2011). Researchers who apply traditional ethnography often attempt to operate in the background as an objective bystander to develop an impartial understanding of their participants (Dharamsi, 2011). For institutional ethnographers, this aim of impartiality is not possible because it relies on one’s ability to remain completely detached from the people one observes (Dharamsi, 2011). Institutional ethnographers are aware that they commit themselves to a certain social relation with the people they are interested in when they begin their projects (Campbell & Gregor, 2004). As Campbell and Gregor (2002) reason, institutional ethnographers’ past experiences and knowledge relate them to the people they are interested in and reveal/establish their location in relation to their collected data. Rather than treating the intuitional ethnographer’s location as a problem of bias, it becomes another way of exposing how knowledge is organized. Smith (2005), herself, writes, “[t]he experiences that the data produces as data may be our own; it may be gained through participation in a workplace or it may be based entirely on interviews” (p. 125).

### Observation as a Starting Point

Institutional ethnographers use observation differently, depending on the purpose of the research study. Diamond (2006), a sociologist, discusses how observation can be like “a starting point on a map, a ‘you are here’ point” (p. 60). Many institutional ethnographers use it to help them realize a problematic for their studies. Smith describes the problematic as “a territory to be discovered” (Smith, p. 41), generally in the early stages of fieldwork. Oftentimes, the problematic is “discovered” when institutional ethnographers notice “disjunctures” or contradictions between official explanations of what is going on and what actually appears to happen (Campbell & Gregor, 2002, Smith, 1990). Once institutional ethnographers have a problematic in mind, their goal is to find other data collection methods, such as interviews, the analysis of texts, or observations of other people, to explain it. The following two examples show how two researchers, Kathleen Benjamin (Benjamin & Rankin, 2014) and Timothy Diamond (2006) (both involved in research in long-term care facilities) used their observations to “discover” the problematics of their studies.

In the first example, Kathleen Benjamin, a registered nurse, used her observations of personal support workers working in a long-term care facility as an entry-point into her doctoral work (Benjamin & Rankin, 2014). She observed mealtimes were very rushed, stressful times in long-term care facilities where she was working and absorbed much of the personal support workers’ time (Benjamin & Rankin, 2014). She noted how the standards in place by the long-term care facilities to provide the residents with a pleasant dining experience actually did the opposite and reduced the time the personal support workers had to support the residents’ physical activity (Benjamin & Rankin, 2014). Benjamin’s problematic emerged from her observations of mealtimes, and her next step was to look for more data that further explicated it (Benjamin & Rankin, 2014).

In the second example, Diamond (2006) reflected on how he completed an institutional ethnography in several long-term care facilities in America. He described how he was surprised to observe an expensive-looking fur coat in a resident’s closet (Diamond, 2006). Diamond (2006) knew this long-term care facility was subsidized and most of the residents came from underprivileged backgrounds. Observing the coat helped Diamond (2006) to see the social relations behind its presence in the resident’s closet. He questioned the resident and learned she once lived in a nice suburb and wore the coat to church with her husband (Diamond, 2006). According to Diamond, “the coat’s journey was a journey of policy in motion” (Diamond, 2006, p. 68). The resident went from her home in the suburbs, to a hospital, to a Medicare long-term care facility, and finally to a subsidized facility after her personal resources were depleted (Diamond, 2006).

## Discussion

Above, we discussed how institutional ethnography differs from traditional ethnography; and how this has implications for how institutional ethnographers use and prioritize their data collections methods, particularly observation. Traditional ethnographers use observation to create “accurate” descriptions of people’s lives in a local setting (Loiselle et al., 2013), they want to answer the question, “what happens?” Institutional ethnographers strive to go beyond descriptions in a local setting to understanding how people unconsciously sustain and support large social institutions through their activation of texts (Turner, 2006). An institutional ethnographer’s goal is not to describe the lives of the people they are interested in—but to map out the social and ruling relations that connect, coordinate, organize, and control them (Turner, 2006).

As institutional ethnographers want to answer the questions “how do things work?” and “how are they put together?” (Kearney et al., 2019), their data collection methods must expand beyond just what people do in a local setting; therefore, it is understandable that institutional ethnographers prioritize interviews and textual analysis over observation. Observation is still arguably an important data collection method and can provide context to how and by whom texts are activated in local settings. Observation makes it possible to confirm whether what people say they do and what they actually do match up. In interviews, institutional ethnographers listen to people describe their actions, usually in past tense—but through observation, institutional ethnographers see peoples’ actions, as they occur, in a particular setting. Institutional ethnographers can use observation to reveal discrepancies between what people self-report and what actually occurs. In addition, conceptual and professional language/rhetoric can conceal what people really do and only direct observation makes such discrepancies known.

Many qualitative approaches use observation as a data collection method, even though there are some issues with this approach, such as the researcher’s presence, bias/selective reporting, time commitment, and accessibility to the field site (Dympna, 2006; Mulhall, 2003). Despite these issues, observations can create a fuller picture for researchers and can be a valuable and useful data collection method. Consequently, institutional ethnographers could consider how these issues pertain to their individual studies, and institutional ethnography as a research approach, before being deterred from using it.

## Conclusion

In conclusion, there are aspects of peoples’ lives that are ruled and organized by institutional guidelines, principles, and regulations, which people or health care professionals may not understand or be able to explain/describe. They may not even be aware how these rules and regulations influence their work or interventions. Including observations as a data collection method creates a contextual picture with interviews and documents/texts of a reality of institutional settings, collaborative approaches, and what actually happens in a local setting. Observation can help institutional ethnographers understand how peoples’ lives are ruled by institutions in ways that they, themselves, may not understand or be able to explain or describe.

## References

[bibr1-10497323211015966] Almagor E. Skinner J. (2013). Ancient ethnography: New approaches. Bloomsbury Publishing.

[bibr2-10497323211015966] Benjamin K. Rankin J. (2014). Reflections of a novice institutional ethnographer. Canadian Journal of Nursing Research, 46(1), 87–101. 10.1177/084456211404600107 29509466

[bibr3-10497323211015966] Bisaillon L. Rankin J. (2012). Navigating the politics of fieldwork using institutional ethnography: Strategies for practice. Forum Qualitative Sozialforschung/Forum: Qualitative Social Research, 14(1), 14. https://www.researchgate.net/publication/288108493_Navigating_the_Politics_of_Fieldwork_Using_Institutional_Ethnography_Strategies_for_Practice

[bibr4-10497323211015966] Campbell M. Gregor F. (2002). Mapping social relations: A primer in doing institutional ethnography. AltaMira Press.

[bibr5-10497323211015966] Campbell M. Gregor F . (2004). Chapter 12: Theory ‘in’ everyday life. In Carro W. ll (Ed.), Critical strategies for social research (pp. 170–181). Canadian Scholar’s Press, Inc.

[bibr6-10497323211015966] Conroy T. (2017). A beginner’s guide to ethnographic observation in nursing research. Nurse Researcher, 24(4), 10–14. 10.7748/nr.2017.e1472 28326918

[bibr7-10497323211015966] Corman M. (2017). Titrating the rig: How paramedics work in and on their ambulances. Qualitative Health Research, 28(1), 47–59. 29103361 10.1177/1049732317739266

[bibr8-10497323211015966] Devault M. (2006). What is institutional ethnography? Social Problems, 53(3), 294–298.

[bibr9-10497323211015966] DeWalt K. DeWalt B. (2002). Participant observation: A guide to fieldwork. AltaMira Press.

[bibr10-10497323211015966] Dharamsi S. (2011). Ethnography: Traditional and criticalist conceptions of a qualitative research method. Canadian Family Physician, 57(3), 378–379. 21402974 PMC3056692

[bibr11-10497323211015966] Diamond T. (2006). Where did you get the fur coat, Fern? Participant observation in institutional ethnography. In Smith D. (Ed.), Institutional ethnography as practice (pp. 45–63). Rowman & Littlefield.

[bibr12-10497323211015966] Duxbury J. Wright K. Hart A. Bradley D. Roach P. Harris N. Carter B. (2010). A structured observation of the interaction between nurses and patients during the administration of medication in an acute mental health unit. Journal of Clinical Nursing, 19(17–18), 2481–2492. 20920076 10.1111/j.1365-2702.2010.03291.x

[bibr13-10497323211015966] Dympna C. (2006). Choosing an appropriate method of data collection. Nurse Researcher, 13(3), 75–92. 10.7748/nr2006.04.13.3.75.c5980 16594371

[bibr14-10497323211015966] Fetterman D. (1989). Ethnography: Step by step. SAGE.

[bibr15-10497323211015966] Fetterman D . (1998). Ethnography: Step by step (2nd ed.). SAGE.

[bibr16-10497323211015966] Guba E. G. Lincoln Y. S . (1994). Chapter 6: Competing paradigms in qualitative research. In Denzin N. Lincoln Y. (Eds.), Handbook of qualitative research (pp. 163–194). Sage Publications, Inc.

[bibr17-10497323211015966] Hammersley M. (1990). What’s wrong with ethnography? The myth of theoretical description. Sociology, 24(4), 597–615.

[bibr18-10497323211015966] Kearney G. Corman M. Hart N. Johnston J. Gormley G . (2019). Why institutional ethnography? Why now? Institutional ethnography in health professions education. Perspectives on Medical Education, 8(1), 17–24. 30742252 10.1007/s40037-019-0499-0 PMC6382621

[bibr19-10497323211015966] Kragelund L. (2013). The obser-view: A method of generating data and learning. Nurse Researcher, 20(5), 6–10. 10.7748/nr2013.05.20.5.6.e296 23687843

[bibr20-10497323211015966] Loiselle C. Profetto-McGrath J. Polit D. Beck C. (2013). Nursing research, principles, & methods. Churchill Livingstone.

[bibr21-10497323211015966] Malachowski C. K. Boydell K. Sawchuk P. Kirsh B. (2016). The “work” of workplace mental health: An institutional ethnography. Society and Mental Health, 6(3), 207–222.

[bibr22-10497323211015966] Marshall C. Rossman G. (1989). Designing qualitative research. SAGE.

[bibr23-10497323211015966] McGibbon E. Peter E. Gallop R. (2010). An institutional ethnography of nurses’ stress. Qualitative Health Research, 20(10), 1353–1378. 20643823 10.1177/1049732310375435

[bibr24-10497323211015966] Montgomery C. Kaul Y. Brodd K. Hellstrom-Westas L. (2020). Structured observation of motor performance in infants: Level and quality associated with later motor development. Acta Paediatrica, 110(1), 307–313. 10.1111/apa.15377 32474945

[bibr25-10497323211015966] Morse J. (2016). Underlying ethnography. Qualitative Health Research, 26(7), 875–876. 27166400 10.1177/1049732316645320

[bibr26-10497323211015966] Mulhall A. (2003). In the field: Notes on observation in qualitative research. Journal of Advanced Nursing, 411(3), 306–313. 10.1046/j.1365-2648.2003.02514.x 12581118

[bibr27-10497323211015966] Ng S. Stooke R. Regan S. Hibbert K. Schryer C. Lingard L. (2013). An institutional ethnography inquiry of health care work in special education: A research protocol. International Journal of Integrated Care, 13, 1–11. 10.5334/ijic.1052 PMC3812304 24179456

[bibr28-10497323211015966] Nightingale F. (1969). Notes on nursing: What it is, and what it is not. Dover. (Original work published 1860)

[bibr29-10497323211015966] O’Connell Davidson J. Layder D . (1994). Methods, sex, and madness. Routledge.

[bibr30-10497323211015966] Quinlan E. (2009). The ‘actualities of knowledge work: An institutional ethnography of multidisciplinary primary health care teams. Sociology of Health and Illness, 31(5), 625–641. 19392938 10.1111/j.1467-9566.2009.01167.x

[bibr31-10497323211015966] Rankin J. (2003). Patient satisfaction: Knowledge for ruling hospital reform. An institutional ethnography. Nursing Inquiry, 10(1), 57–65. 12622805 10.1046/j.1440-1800.2003.00156.x

[bibr32-10497323211015966] Rankin J. Campbell M. (2006). Managing to nurse: Inside Canada’s health reform. University of Toronto Press.

[bibr33-10497323211015966] Rankin J. Campbell M. (2009). Institutional ethnography (IE), nursing work and hospital reform: IE’s cautionary analysis. Forum Qualitative Sozialforschung/Forum: Qualitative Social Research, 10(2), 8. https://www.qualitative-research.net/index.php/fqs/article/view/1258

[bibr34-10497323211015966] Rankin J. M . (2017). Conducting analysis in institutional ethnography: Analytical work prior to data collection. International Journal of Qualitative Methods, 16. 10.1177/1609406917734484

[bibr35-10497323211015966] Rankin J . (2017). Conducting analysis in institutional ethnography: Guidance and cautions. International Journal of Qualitative Methods, 16. https://doi-org.proxy.hil.unb.ca/10.1177/1609406917734472

[bibr36-10497323211015966] Rashid M. Caine V. Goez H. (2015). The encounters and challenges of ethnography as a methodology in health research. International Journal of Qualitative Methodology in Health Research, 14(5). 10.1177/1609406915621421

[bibr37-10497323211015966] Salmon J. (2015). Using observational methods in nursing research. Nursing Standard, 29(45), 36–41. 10.7748/ns.29.45.36.e8721 26153969

[bibr38-10497323211015966] Schmuck R. (1997). Practical action research for change. IRI/Skylight Training & Publishing.

[bibr39-10497323211015966] Smith D. (1990). Texts, facts, and femininity: Exploring the relations of ruling. Routledge.

[bibr40-10497323211015966] Smith D. (2001). Texts and the ontology of organizations and institutions. Studies in Cultures, Organizations and Societies, 7(2), 159–198.

[bibr41-10497323211015966] Smith D. (2005). Institutional ethnography: A sociology for people. AltaMira Press.

[bibr42-10497323211015966] Smith D. (2006). Institutional ethnography as practice. Rowman & Littlefield.

[bibr43-10497323211015966] Taber N . (2010). Institutional ethnography, autoethnography, and narrative: An argument for incorporating multiple methodologies. Qualitative Research, 10(1), 5–25.

[bibr44-10497323211015966] Townsend E. Langille L. Ripely D. (2003). Professional tension in client-centred practice: Using institutional ethnography to generate understanding and transformation. The American Journal of Occupational Therapy, 57(1), 17–28. 12549887 10.5014/ajot.57.1.17

[bibr45-10497323211015966] Tummons J. (2017). Institutional ethnography, theory, methodology, and research: Some concerns and some comments. In Reid J. Russell L. (Eds.), Perspectives on and from institutional ethnography studies in qualitative methodology (Vol. 15, pp. 147–162). Emerald Publishing. 10.1108/S1042-319220170000015003

[bibr46-10497323211015966] Tummons J. Macleod A. Kits O . (2015). Ethnographies across virtual and physical spaces: A reflexive commentary on a live Canadian/UK ethnography of distributed medical education. Ethnography and Education, 10(1), 107–120.

[bibr47-10497323211015966] Turner S. (2006). Mapping institutions as work and texts. In Smith D. (Ed.), Institutional ethnography as practice (pp. 139–161). Rowman & Littlefield.

